# Predicting Adolescent Depression: The Interrelated Roles of Self-Esteem and Interpersonal Stressors

**DOI:** 10.3389/fpsyg.2019.00565

**Published:** 2019-03-15

**Authors:** Caterina Fiorilli, Teresa Grimaldi Capitello, Daniela Barni, Ilaria Buonomo, Simonetta Gentile

**Affiliations:** ^1^Dipartimento di Scienze Umane, Libera Università Maria SS. Assunta di Roma, Rome, Italy; ^2^Ospedale Pediatrico Bambino Gesù, Rome, Italy; ^3^Consorzio Universitario Humanitas, Rome, Italy

**Keywords:** adolescents, depression, self-esteem, interpersonal relationship, stressor sources

## Abstract

Depression in adolescents can lead to social and educational impairment and is a major risk factor for suicide and substance misuse. Thus, predicting and preventing this disorder are extremely important. The current study aimed to analyze the contribution of adolescents’ self-esteem (i.e., quality of interpersonal relationships, control of life events, and management of negative emotions) and interpersonal stressor sources (relationships with parents, teachers, classmates and friends) in predicting several depression manifestations (i.e., depressed mood, sense of inadequacy, and insecurity). Participants were 182 Italian pre-adolescents and adolescents, aged 10–14 years, were recruited from three Italian schools. They were asked to complete a self-report questionnaire. Results showed that self-esteem was a major factor to be considered in adolescents’ depression. In particular, adolescents’ perception of negative emotion management was the most important protective factor against depression manifestations. Conversely, sources of interpersonal stressors contributed only marginally to depression. Among these, problems with parents and friends increased adolescents’ depressed mood, while troubles with classmates impacted on their sense of inadequacy and insecurity. Implications of these results for positive practices which could enhance adolescents’ self-esteem and further expansions of the study are discussed.

## Introduction

Adolescents are at higher risk to develop depression than younger children (Costello et al., 2011). Depression incidence, notably in girls, rises sharply after puberty and is often associated with morbidity and suicide risk (Rhew et al., 2010; Salk et al., 2016). Moreover, depression has detrimental effects on adolescents’ social and academic functioning (Verboom et al., 2014). In their meta-analysis on the prevention of depression in children and adolescents, Horowitz and Garber (2006) highlighted the important role played by implementing mastery learning and behavioral management programs to prevent depression in school-age children.

Preventive actions addressing self-esteem may have a key role to play in reducing these risks. Self-esteem is the affective component of self-concept, as it concerns people’s global appraisal of their positive or negative value, depending on how they estimate their value in different life domains (Harter, 1999). High self-esteem is related to several well-being conditions, such as high happiness and life satisfaction (Duffy et al., 2014), as well as low anxiety, depression, and loneliness (Cacioppo et al., 2009). More specifically, adolescents with low self-esteem are at higher risk for depression. Low levels of self-esteem have been found to predict depressive symptoms in a time-frame ranging from 3 months to 10–15 years later on (Trzesniewski et al., 2006). Interestingly, Orth et al. (2008), in two samples of early and late adolescents, showed that self-esteem predicted depression after 6 years, while depression did not predict subsequent levels of self-esteem.

During adolescence, interpersonal relationships with parents, teachers, and friends may help and support their development. The ability to feel close to and accepted by parents (Mattanah et al., 2011; Lundervold et al., 2013), teachers, and peers (Birkeland et al., 2014) is strongly related to self-esteem. The significant impact of interpersonal relationships on adolescents’ self-esteem may be due to several factors, including the increasing ability to form meaningful psychological relationships with others and the possibility to learn about oneself and one’s own functioning in specific contexts (Nasir and Munaf, 2010; Compare et al., 2013; Buonomo et al., 2017). At the same time, research has shown that relationships can also be a common source of stress in adolescence (Compas et al., 2017). As relationships become more salient, they may generate negative events which, in turn, may be more predictive of a general maladjustment (Hankin et al., 2007). Effectively, interpersonal domains serve as sources of psychosocial stress in adolescence social network stress relationships outside the family increase the possibility of loss, rejection, and conflict. Recently, Rueger et al. (2016) in their systematic meta-analysis of the literature covering relations between social support and depression have shown that family support followed by school support. More specifically, social support may play a stress-buffering effect on children with high-stress conditions.

The association between stress, self-esteem, and depression is easily predictable when considering, on the one hand, the effects of self-esteem on adjustment and well-being and on the other hand, the detrimental association between self-esteem and interpersonal stressors (Babore et al., 2016). Indeed, when individuals perceive their interpersonal relationships as sources of stress, they experience life events as unpredictable or overwhelming (Rudolph, 2002). Moreover, some studies have shown that parents, teachers, classmates, and friends may significantly stress adolescents out, heightening the risk of developing depressive symptoms (Hutcherson and Epkins, 2009). However, to the best of our knowledge, no studies have addressed the relative contribution of individual factors in this context. Addressing the specific role of these variables could help identify protective factors to assist practitioners and educators in their task.

The main limitation of previous studies was the lack of focus on the interrelated roles of self-esteem and sources of interpersonal stressors in adolescent depression. We addressed two main exploratory questions: “How much can adolescents’ self-esteem and interpersonal stressors explain their depressive symptoms?” and “Which aspects of self-esteem and which interpersonal stress sources are the most important predictors of adolescents’ depressive symptoms?”

## Materials and Methods

### Participants and Procedure

The study included 182 Italian pre-adolescents and adolescents (92 girls, 50.55%, and 90 boys, 49.45%), aged 10–14 years (*M* = 12.7, *SD* = 0.875), with no psychological diagnosis. Participants were recruited from three schools located in Rome, Italy. They, as well as their parents, were informed in advance about the main objectives of the research and that participation was free and voluntary. Children whose parents consented to their participation filled in a self-report and anonymous questionnaire in their classrooms during school hours. This study was approved by the Scientific Board of the LUMSA University.

### Measures

#### Depression

The Self-Administered Psychiatric Scales for Children and Adolescents-Depressive Scale (SAFA-D; Cianchetti and Fancello, 2001) was used for a total of 21 items with a total score ranging from 21 to 144. Respondents were asked to answer using a three-point Likert scale (1 = true to 3 = false). In the current study, we used the following scales: Depressed mood composed by seven items (α = 0.89), Sense of inadequacy composed by seven items (α = 0.78), and Insecurity composed by seven items (α = 0.82).

#### Self-Esteem

Three subscales of the Multidimensional Self-Concept Scale (MSCS, Bracken, 1992; It. ad. Mazzeo, 2003) were used and respondents were asked to answer using a four-point Likert scale (1 = strongly agree to 4 = strongly disagree). MSCS was composed by 75 items with a total score ranging from 75 to 300. In the current study, we used the following scales: Interpersonal Relationships composed by 25 items (α = 0.85), Control of life events composed by 25 items (α = 0.78), and Management of negative emotions composed by 25 items (α = 0.78).

#### Sources of Interpersonal Stressors

Adolescents were asked to answer using a four-point Likert scale (1 = strongly agree to 4 = strongly disagree) to what extent parents, classmates, teachers, and friends outside the class were the source of their problems.

### Data Analysis

Preliminarily, we described the study variables in terms of means and standard deviations and measured their associations using Pearson correlations. Second, we conducted three separate multiple linear regressions (MRs), with depressed mood, sense of inadequacy, and insecurity as criterion variables. We estimated the overall *R*^2^ and determined the statistical significance of individual regression coefficients which indicate the extent to which the criterion variable would change based on a given increase in one predictor while the other predictors remained constant. However, when predictors are correlated – as likely in the case of the multidimensional predictors (e.g., self-esteem) – regression coefficients could be inadequate reflections of predictor importance, because all the predictors may interact with each other and simultaneously weight the criterion variable (Barni, 2015). We therefore combined MR with relative weight analysis (RWA), which uses a variable transformation approach to address the issue of correlated predictors. RWA focuses on the proportionate contribution each predictor makes to *R*^2^, considering both its unique relation with the criterion and its relation when combined with other predictors (i.e., relative contribution) (Johnson, 2000).

## Results

Table 1 presents the descriptive statistics of the study variables and the Pearson correlation coefficients, many of which were statistically significant. Specifically, self-esteem dimensions (i.e., interpersonal relationships, control of life events, and management of negative emotions) show the highest correlation values with depression (Pearson coefficients ranging from -0.65 to -0.76, *p* < 0.01). At the same time, sources of interpersonal stress (i.e., parents, teachers, classmates, friends) show significant, but lower correlation values (Pearson coefficients ranging from -0.15 to -0.27, *p* < 0.01). The multiple regression models were significantly predictive of depressed mood, sense of inadequacy, and insecurity (Table 2). Inspection of β weights revealed that self-esteem was the strongest predictor of adolescent depression. More precisely, the higher the scores were on interpersonal relationships and management of negative emotions, lower was the risk of feeling depressed, inadequate, and insecure. Additionally, the stronger the perception of control over events was, lower was the sense of inadequacy.

**Table 1:** Means (*M*), standard deviations (*SD*), and Pearson correlation coefficients (*N* = 182).

Variables	*M*(*SD*)	1	2	3	4	5	6	7	8	9	10
1. Age	12.07 (0.84)	-									
*Self-esteem*											
2. Interpersonal relationships	73.35 (11.17)	0.02	-								
3. Control of life events	73.71 (9.64)	-0.04	0.70**	-							
4. Management of neg. emotion	71.21 (12.74)	0.07	0.72**	0.76**	-						
*Sources of interper. stressors*											
5. Parents	2.09 (0.97)	0.09	-0.23**	-0.21**	-0.25**	-					
6. Teachers	2.16(0.87)	0.04	-0.10	-0.18*	-0.17*	0.22**	-				
7. Classmates	1.83 (0.87)	-0.06	-0.25**	-0.15*	-0.18*	0.08	0.14	-			
8. Friends	1.85 (0.89)	0.08	-0.07	-0.04	-0.07	0.12	0.01	0.24**	-		
*Depression*											
9. Depressed mood	3.24 (3.47)	-0.07	-0.65**	-0.56**	-0.75**	0.22**	0.09	0.15*	0.20**	-	
10. Sense of inadequacy	3.03 (3.63)	-0.01	-0.71**	-0.69**	-0.76**	0.15*	0.08	0.21**	0.12	0.75**	-
11. Insecurity	6.68 (3.58)	-0.01	-0.62**	-0.59**	-0.69**	0.21**	0.10	0.28**	0.17*	0.64**	0.66^∗∗^


*Note. ^∗∗^*p* < 0.01, ^∗^*p* < 0.05*.

**Table 2:** The importance of adolescents’ self-esteem and sources of interpersonal stressors in predicting depression: multiple regression analysis (MR) and relative weight analysis (RWA) results.

Predictors	Depressed mood					Sense of inadequacy					Insecurity				
		MR		RWA		MR		RWA			MR		RWA		
	β	*t*	*p*	Raw importance	Rescaled importance (%)	β	*t*	*p*	Raw importance	Rescaled importance (%)	β	*t*	*p*	Raw importance	Rescaled importance (%)
Gender	0.22	3.07	0.002	0.02	3.5	0.21	2.88	0.004	0.02	2.8	0.34	4.74	0.000	0.07	11.4
Age	-0.05	-0.62	0.534	0.00	0.6	0.02	0.29	0.768	0.00	0.1	0.03	0.49	0.624	0.00	0.2
*Self-esteem*															
Interpersonal relationships	-0.28	-3.83	0.000	0.18	28.0	-0.29	-4.18	0.000	0.20	30.5	-0.22	-2.87	0.006	0.14	24.6
Control of life events	0.10	1.33	0.186	0.10	16.7	-0.19	-2.50	0.013	0.18	27.1	-0.12	-1.46	0.144	0.12	21.2
Management of neg. emotion	-0.62	-7.45	0.000	0.27	43.0	-0.40	-5.11	0.000	0.23	35.3	-0.36	-4.13	0.000	0.18	31.7
*Sources of interp. stressors*															
Parents	0.18	2.53	0.012	0.02	2.4	0.11	1.52	0.131	0.01	0.9	0.16	2.25	0.026	0.01	2.1
Teachers	0.04	0.60	0.551	0.00	0.4	0.04	0.52	0.604	0.00	0.3	0.04	0.59	0.555	0.00	0.4
Classmates	0.07	1.00	0.321	0.01	0.8	0.17	2.26	0.025	0.01	2.0	0.23	3.23	0.001	0.03	5.9
Friends	0.16	2.17	0.031	0.03	4.6	0.05	0.69	0.488	0.01	1.0	0.08	1.14	0.255	0.02	2.5
*R*^2^				0.63	100				0.66	100				0.57	100
*F*(9,181)	31.01**					36.53**					25.50**				


*Note. Rescaled importance was computed by dividing the relative weights by the total *R*^*2*^ and multiplying by 100. ^∗∗^*p* < 0.001.*

Among the sources of interpersonal stressors, problems with parents were a risk factor for adolescents’ depressed mood and insecurity, problems with classmates for sense of inadequacy and insecurity, and problems with friends for depressed mood and sense of inadequacy. Negative relationship with teachers, however, did not help predict any adolescent depression dimension. Girls showed higher levels of depressed mood, sense of inadequacy, and insecurity than boys did, with the largest gender difference found in insecurity. Conversely, adolescents’ age was not related to the criterion variables.

The analysis through RWA of the relative contributions of each predictor in explaining adolescent depression largely confirmed the importance of self-esteem in accordance with the regression results (Table 2). Adolescents’ ability to manage negative emotions was the most important predictor in the context of other predictors, followed by interpersonal relationships. Interestingly, RWA evaluated the contribution of adolescents’ ability to control life events in predicting depression: it accounted for 16.7% of depressed mood variance, for 27.1% of inadequacy variance, and for 21.2% of insecurity variance. Probably because of the high correlation between this variable and interpersonal relationships (*r* = 0.70, *p* < 0.01) and management of negative emotions (*r* = 0.76, *p* < 0.01), the regression coefficients seemed to indicate that control of life events did not contribute significantly to depressed mood and insecurity.

According to RWA results, the three sources of interpersonal stressors contributed only marginally to depression. Among these predictors, problems with classmates were the most important predictors of insecurity (5.9% of the explained variance) and sense of inadequacy (2.0% of the explained variance); problems with friends were the most important in the case of depressed mood (4.6% of the explained variance).

## Discussion

The aim of the current study was to analyze the importance of self-esteem and interpersonal stressor sources in predicting different adolescent depression manifestations. Overall, our results showed that self-esteem and sources of interpersonal stressors play a large role in the development of depressed mood as well as sense of inadequacy and insecurity in adolescents.

The strongest predictor of adolescent depression was self-esteem (i.e., perception regarding interpersonal relationships, negative emotions management, and control over life events). We found that, of all the predictors, the perception of negative emotion management was the most important protective factor against depressed mood, inadequacy, and insecurity. Adolescents with low self-esteem feel incompetent and worthless; they then try to manage the negative emotions arising from these beliefs but in a dysfunctional way, thus becoming even more stressed (Eisenbarth, 2012). Coherently, high levels of conflicts, like anger and rejections, contribute significantly to the persistence of depressive symptoms (Vulić-Prtorić and Macuka, 2006). With regard to interpersonal relationships, the four sources of stress we analyzed (i.e., parents, teachers, classmates, and friends) contributed only marginally to depression.

Among these, problems with classmates, friends, and parents impacted on depression, similarly to the findings of previous studies (Ng et al., 2007). Previous findings indeed showed that young adolescents are at higher risk when they feel high criticism and rejection by peers (i.e., friends and classmates) (Passiatore et al., 2017). Moreover, parental warmth and emotional support may be an important factor in protection against depression in children and adolescents (Capitello et al., 2016). According to Wang and Sheikh-Khalil (2014), some forms of parental involvement in their children’s lives may positively affect their general self-esteem (e.g., supportive parent–adolescent communication at home), which, in turn, may protect adolescents against depressive symptoms (Flouri and Buchanan, 2003; Bean et al., 2006).

Even so, our results showed that parents and friends contribute to depressed mood, while classmates influence adolescents’ sense of inadequacy and insecurity. While several studies showed that non-supportive, negative relationships with parents and close friends heighten depressive risks (La Greca and Harrison, 2005; Wang and Sheikh-Khalil, 2014), the impact of classmates on insecurity was studied less. Prinstein and La Greca (2002) showed that adolescents affiliated with a low-status group at school are more insecure as regards their behavior, appearance, and social and athletic abilities than peers affiliated with more popular and accepted groups.

Conversely, it emerged from our study that problems with teachers did not explain the dimension of adolescent depression. This result, however, requires deeper analysis in additional studies in view of the well-known relevance for adolescents’ well-being of the school-life context and relationships with its actors (Fiorilli et al., 2017a,b).

Finally, our results confirmed that an adolescents’ gender is associated with depression symptoms as reported in previous studies (Girgus and Yang, 2015).

Several questions emerging from our findings need further research in order to overcome the caveats of our study. First, collection of data concerning adolescents’ academic achievements may support analysis regarding adolescents’ adjustment in the school context and may also show their impact on the adolescents’ reputation within the family context (Moreira et al., 2015). Second, multi-informant methods would be particularly helpful and supportive in studies which focus on interpersonal relationships. Third, multiple measures should be considered for the future, with specific attention to the sources of stress.

Furthermore, due to the cross-sectional research method, the effective developmental pathways among studies variables remain unexplored. Finally, with regard to the gender differences which emerged from the current study, an interesting perspective is presented by other studies which suggest that gender differences in self-report measures are due to the high level of awareness of females compared to their male peers.

## Data Availability

The datasets for this manuscript are not publicly available because of local legal and privacy restrictions (Italian Data Protection Code – Legislative Decree no. 196/2003). All datasets generated for this study are included in the manuscript and/or the supplementary files.

## Ethics Statement

The authors assert that all procedures contributing to this work comply with the ethical standards of the relevant national and institutional committees on human experimentation. All participants gave written informed consent in accordance with the Declaration of Helsinki. The study was approved by the Scientific Board of LUMSA University.

## Author Contributions

CF designed and carried out the study and contributed to the analysis of the results and to the writing of the manuscript. TGC designed and carried out the study, collected data, and contributed to the writing of the manuscript. DB contributed to the analysis of the results and to the writing of the manuscript. IB collected data and contributed to the writing of the manuscript. SG supervised the study design and the manuscript draft.

## Conflict of Interest Statement

The authors declare that the research was conducted in the absence of any commercial or financial relationships that could be construed as a potential conflict of interest.
